# Prognosis of non-vital incisors after apexification using bioceramics: a retrospective study

**DOI:** 10.1007/s40368-024-00915-5

**Published:** 2024-06-05

**Authors:** F. S. Anjum, I. J. Brusevold, T. I. Wigen

**Affiliations:** Department of Paediatric Dentistry, Behavioural Science and Forensic Dentistry, Institute of Clinical Dentistry, University of Oslo, Box 1109, Blindern, 0317 Oslo, Norway

**Keywords:** Apexification, Biodentine, MTA, Open apices, Pulp necrosis

## Abstract

**Purpose:**

To evaluate the outcome of apexification using bioceramics in immature permanent teeth, and to study the factors influencing treatment outcome and frequency of spontaneous cervical root fractures.

**Methods:**

Forty-six children with 51 non-vital permanent incisors treated with a bioceramic apical plug were included. Mean age at pulp necrosis was 9.8 (SD 2.2) years and mean follow-up time was 3.3 (SD 2.4) years. Data were extracted from dental records and included stage of root development, presence of external root resorptions, clinical signs of infection, size of periapical lesion, type and placement of bioceramic plug, and spontaneous root fracture. Treatment outcome was assessed using periapical index (PAI-score) and presence of clinical symptoms. The statistical analyses were conducted using IBM SPSS Statistics for Windows, version 28 (IBM Corp., Armonk, N.Y., USA). Data were cross-tabulated and tested with chi-square statistic.

**Results:**

Biodentine™ was used as apical plug in 78.4% of the teeth and MTA in 21.6%. Complete healing or signs of healing was reported in 86.3% of the teeth, whilst seven teeth (13.7%) were non-healed. All non-healed teeth had preoperative clinical signs of infection. No difference was found in stage of root development, presence of root resorption, and type and placement of bioceramic plug in healed and non-healed teeth (*p* > 0.05). Five teeth (9.8%) exhibited root fracture 1–6 years after treatment and all had very immature root development. Type of bioceramic or external root resorption was not associated with spontaneous root fracture.

**Conclusion:**

Apexification using bioceramics showed favourable prognosis in immature permanent teeth. Very immature teeth were at risk of root fracture after apexification with bioceramics.

## Introduction

Traumatic dental injury is the most common etiological factor for pulp necrosis in immature anterior permanent teeth (Andreasen and Pedersen 1985; Hecova et al. 2010). Other factors such as dental caries or dental developmental defects may also cause pulp necrosis in immature teeth (Alani and Bishop 2008). A vital pulp is necessary for the development and maturation of the root. If vitality is lost, the maturation process will arrest and, depending on the patient’s age, leave the tooth with a wide root canal, fragile dentinal walls and an open apex (Trope 2010). In immature teeth, root canal treatment is complicated by the lack of an apical constriction against which to condense and contain a root filling. In such cases, the traditional treatment has been apexification with long-term use of calcium hydroxide to stimulate apical hard tissue formation, allowing canal obturation with gutta-percha (GP) (Kerekes et al. 1980).

Long-term calcium hydroxide apexification technique has been shown to be very successful (Kerekes et al. 1980; Cvek 1992), but has disadvantages such as long treatment time with multiple appointments which requires a high degree of patient compliance (Dominguez Reyes et al. 2005). There is also risk of temporary restoration loss and subsequent re-infection (European Society of Endodontology 2006). The evidence regarding negative side effects of long-time use of calcium hydroxide, i.e. weakening of root dentine and increased risk of cervical root fracture are inconclusive (Yassen and Platt 2013; Bonte et al. 2015;Best et al. 2021). One study including 59 incisors in adults reported no root fractures in teeth treated with long-term calcium hydroxide (Best et al. 2021). Others have reported that immature permanent teeth have a higher rate of spontaneous cervical root fractures than mature teeth, ranging from 77% in teeth with the least root development to 28% in teeth with the most developed roots (Cvek 1992). Cervical root fractures have also been associated with cervical defects following healing of external root resorptions (Cvek 1992).

The use of bioceramics has become an alternative calcium hydroxide for creating an apical barrier in non-vital immature permanent teeth (Holden et al. 2008; Mente et al. 2013). The rationale behind apexification is to establish an apical stop enabling immediate root canal filling. Clinical studies have reported healing of teeth with open apices after placing an apical plug with mineral trioxide aggregate (MTA) (Simon et al. 2007; Ree and Schwartz 2017). MTA is a calcium silicate-based material developed by the modification of Portland cement. It has good biocompatibility and sealing properties with low cytotoxicity. In addition, the sealing properties of MTA are not affected by moisture during treatment. MTA, like calcium hydroxide, stimulates apical hard tissue formation (Parirokh and Torabinehad 2010a, b; Torabinejad and Parirokh 2010). However, well-known disadvantages are tooth discoloration and a long setting time requiring an extra appointment (Możyńska et al. 2017).

Recently, a new biomaterial Biodentine™ (Septodont, Saint Maur des Faussés, France) was developed for use as a dentine substitute. Biodentine™ is a calcium silicate-based product which has shown favourable physical, biological, and handling properties (Rajasekharan et al. 2018). The material is suitable as alternative to MTA in apexification. It has a reduced setting time (10–12 min) compared to MTA, allowing the canal to be obturated with GP at the same visit (Bachoo et al. 2013). Biodentine™ is frequently used in paediatric dental practice, but there is lack of clinical studies on the success of apexification of immature permanent incisors using this material.

The aim of this retrospective study was to evaluate outcome of apexification using bioceramics in immature permanent teeth, and to study factors influencing treatment outcome and frequency of cervical root fractures.

## Materials and methods

This manuscript was written in accordance with the **ST**rengthening the **R**eporting of **OB**servational **S**tudies in **E**pidemiology (STROBE) guidelines.

### Participants

All patients referred for treatment of non-vital anterior permanent teeth to the Department of Pediatric Dentistry, Behavioral Science and Forensic Dentistry at University of Oslo from 2008 to 2021 were included in the study. The inclusion criteria were permanent anterior teeth diagnosed with pulp necrosis, apexification with a bioceramic apical plug and age under 18 years. In total, 48 patients met the inclusion criteria. Two patients were lost to follow-up and the study group comprised 46 patients (34 boys and 12 girls) with 51 teeth.

### Data collection

Postgraduate dentists under supervision of a specialist in paediatric dentistry performed treatments and follow-ups. Data were extracted from dental records. Preoperative data included gender, tooth, child age and stage of root development at pulp necrosis, aetiology of necrosis, clinical signs of infection, radiographic evidence of pulp necrosis and root resorption.

The stage of root development prior to treatment was classified according to Moorrees classification (Moorrees et al. 1963) and divided into three groups of increasing tooth maturity: Very immature (ranged from initial root formation to root length completed with parallel ends), immature (full root length and apical foramen half closed) or mature (full root length and closed apex). Five teeth had sustained external apical root resorptions (three teeth) or root fractures (two teeth) which left the teeth with open apices and short roots prior to apexification, and these were classified as very immature. These five teeth were excluded from the analyses of spontaneous root fracture. Root development was categorised as very immature or immature in the analyses as no teeth had mature root development.

Aetiology of pulp necrosis was assessed as dental trauma or developmental defects such as invagination.

Clinical signs of infection included sensitivity to percussion and palpation, sinus tract or abscess, and was classified as present or absent in the analyses. Pulp necrosis was verified using an electric pulp tester (Dahlin Electronic Pulp Tester) and cold pulp sensibility test (Endo Ice; Roeko, 71 Langenau, Germany; Endo Frost, Coltène Whaledent Roeko, Germany).

Radiographic evidence of pulp necrosis was reported as periapical lesion or arrested root development. Root resorption included infection related root resorption and replacement resorption and were classified as present or absent.

Periapical status at apexification and at follow-ups was scored on digital radiographs using the periapical index (PAI) (Ørstavik et al. 1986). PAI classifies radiographically the tooth’s periapical status on a grade from 1 (healthy) to 5 (severe apical periodontitis). Before scoring the radiographs, the examiner (FSA) was calibrated using the PAI kit where 100 reference radiographs, not related to this study, were evaluated and graded on a scale from 1 to 5. Two score sets were produced 10 days apart and was compared to a standard established by Ørstavik et al. to determine agreement (Ørstavik et al. 1986). Kappa values showed substantial agreement for inter-examiner (κ = 0.78) and intra-examiner (κ = 0.79) reproducibility against the standard. All periapical radiographs were presented on a computer screen and blindly evaluated in a dark room by the first author (FSA). The periapical area was graded on a scale from 1 to 5 and compared with five reference radiographs. Any uncertainty regarding PAI-score for a particular tooth was discussed with the other authors (TW and IJB) and resulted in joint assessment.

Treatment was performed according to a standard protocol of root canal treatment used at The Department of Pediatric Dentistry, Behavioral Science and Forensic Dentistry at University of Oslo, based on the recommendations from European Society of Endodontology (European Society of Endodontology 2006). Teeth were isolated with rubber dam after injection of local anaesthesia (Septocaine (Articaine) 40 mg/ml + 5 µg/ml, Septodont Inc, New Castle, DE). Chemo-mechanical debridement was achieved using endodontic files and irrigation with 1% sodium hypochlorite (NaOCL) and 17% ethylenediaminetetraacetic acid (EDTA). Calcium hydroxide paste was used as an inter-appointment medicament. At the time of obturation, Biodentine™ (Septodont, Saint Maur des Faussés, France), white MTA ProRoot® (Dentsply, Tulsa Dental, Tulsa, OK, USA) or white MTA Angelus® (Angelus Dental Solutions, Londrina, Parana, Brazil) was placed in the apical portion of the root using either a Micro-Apical Placement System (Dentsply Maillefer, Vevey Switzerland) or an endodontic plugger. A periapical radiograph was taken to confirm the correct position of the bioceramic plug. The root canal was obturated with thermo-plasticized GP (Obtura Spartan, Fenton, MO, USA) or with GP placed with cold lateral condensation technique (one tooth) with AH Plus® sealer (Dentsply Sirona Endodontics) and IRM® (Dentsply) in the coronal portion of the root. Obturation with GP was conducted in the same appointment as apexification when Biodentine was used, whilst in a second appointment when MTA was used for apexification. Composite resin material was used to seal the access cavity.

Follow-up data included the presence or absence of clinical symptoms and periapical status. Any pain or soft tissue lesion associated with the tooth was defined as a clinical symptom. Tenderness to percussion or palpation was considered a subjective symptom and accepted if unaccompanied by any other clinical signs or symptoms. Outcomes were recorded at follow-ups and were based on radiographic outcome (PAI-score) and by criteria from Holden et al. (Holden et al. 2008). The radiographic outcome was considered healed when PAI-score was 1 or 2, or non-healed when PAI-score was 3, 4 or 5. The outcome according to Holden’s criteria included clinical symptoms in addition to radiographic measures as shown in Table 1. In the analyses, the radiographic outcome and outcome according to Holden were dichotomized as healed (healed or healing) or non-healed.

**Table 1:** Criteria used to determine healing outcome according to Holden (Holden et al. 2008)

Outcome	PAI	Clinical symptoms
Healed	PAI 1 or 2	None present
Healing	PAI 3 or 4, with improved score at follow-up	None present
Non-healed	PAI 1–5	Present
Non-healed	PAI 3–5, with score not improved or worse at follow-up	None present

*PAI* periapical index

The position of the bioceramic plug was scored as ideal (plug was positioned in the apical portion of the root) or non-ideal (overfilled or underfilled) at apexification. The radiopacity of the bioceramic material was assessed on radiographs at apexification and at last follow-up and categorised as no change of radiopacity or change of radiopacity. A spontaneous root fracture was recorded when it was seen and not caused by a second injury. The five teeth that had undergone root fracture or had external root resorption before treatment were excluded from these analyses.

### Statistical analyses

The statistical analyses were conducted using IBM SPSS Statistics for Windows, version 28 (IBM Corp., Armonk, N.Y., USA). Data were cross-tabulated and tested with chi-square statistics, and presented as frequency, mean and standard deviation. The level of significance was set at 5%.

## Results

The included teeth were 45 maxillary central incisors (88%), five lateral incisors (9.8%) and one mandibular central incisor. Five patients had more than one tooth treated with apexification. Trauma was the most common etiological factor for pulp necrosis, and almost half (43.1%) of the included teeth had combination injuries: trauma to hard tissue in combination with luxation injury. The most frequent single luxation injuries were avulsion (19.6%), lateral luxation (11.8%) and intrusion (7.8%).

Mean age at pulp necrosis was 9.8 (SD 2.2) years. Half of the teeth (52.9%) had very immature root development (Table 2). More than half (60.8%) had signs of infection present before endodontic treatment started, and one-third (33.3%) showed signs of external root resorption. Calcium hydroxide dressing was used in all teeth prior to apexification. Mean treatment time was 8.2 (SD 8.4) months with half of the teeth treated for less than 6 months. Ten teeth had dressing longer than 12 months. Periapical periodontitis with PAI scores 3 or 4 was diagnosed in twenty (39.2%) of the teeth prior to apexification. Biodentine™ was used as apical barrier in most teeth (78.4%).

**Table 2:** Root development, signs of infection, root resorption, type of bioceramic and placement of bioceramic plug for all teeth and comparison of healed and non-healed teeth (n = 51)

	All (*n* = 51)		Healed (*n* = 44)		Non-healed (*n* = 7)		*P*-value
	%	(*n*)	%	(*n*)	%	(*n*)	
*Root development*							
Very immature	52.9	(27)	50.0	(22)	71.4	(5)	ns
Immature	47.1	(24)	50.0	(22)	28.6	(2)	
*Signs of infection*							
Present	60.8	(31)	54.5	(24)	100.0	(7)	< 0.05
Absent	39.2	(20)	45.5	(20)	0	(0)	
*Root resorption*							
Present	33.3	(17)	34.1	(15)	28.6	(2)	ns
Absent	66.7	(34)	65.9	(29)	71.4	(5)	
*Type of bioceramic*							
Biodentine™	78.4	(40)	77.3	(34)	85.7	(6)	ns
MTA	21.6	(11)	22.7	(10)	14.3	(1)	
*Bioceramic plug*							
Ideal	58.8	(30)	56.8	(25)	71.4	(5)	ns
Non-ideal	41.2	(21)	43.2	(19)	28.6	(2)	

Ideal placement of bioceramic plug was found in thirty (58.8%) teeth, whilst six (11.8%) teeth were overfilled and 15 (29.4%) teeth underfilled. A higher proportion of non-ideal plugs were found in teeth treated with MTA compared to Biodentine™ but the difference was not statistically significant (63.6% vs. 35.0%, *p* = 0.087).

The follow-up period after final obturation ranged from 0.6 to 10.5 years with mean 3.3 years (SD 2.4). Only three teeth had follow-up less than one year.

The radiographic outcome based on PAI-score at last follow-up showed that 45 (88.2%) of the teeth had reduced or unchanged PAI-score and were classified as radiographically healed, whilst six teeth (11.8%) had increased PAI-score at follow-up. According to the outcome criteria by Holden et al. (Holden et al. 2008), forty-three (84.3%) of the teeth were asymptomatic with PAI-score 1 or 2 and were healed, whereas one tooth (2%) was asymptomatic with a decrease in PAI-score and considered healing. Three teeth showed increased replacement resorption after apexification but were classified as healed and functioned as temporary space maintainers until replacement. Seven teeth (13.7%) were classified as non-healed, of which two teeth showed signs of persistent infection (Table 3).

**Table 3:** Description of the seven teeth classified as non-healed according to Holden (Holden et al. 2008)

Clinical symptoms	Radiographic description	PAI-score	(*n*)
Absent	Bioceramic extruded out of apex, periapical radiolucency present	3	(2)
Absent	Periapical radiolucency decreased, no change in PAI-score	3	(1)
*Present*			
Pain and infection		4	(2)
Orthodontic movement or short root anomaly		1	(2)

*PAI* periapical index

Table 2 shows teeth classified as healed or non-healed and association with pre- and perioperative findings. All seven teeth classified as non-healed had preoperative signs of infection. Neither root development, presence of root resorption, type of bioceramic nor placement of the bioceramic plug significantly influenced healing outcome (*p* > 0.05).

**Fig. 1:**
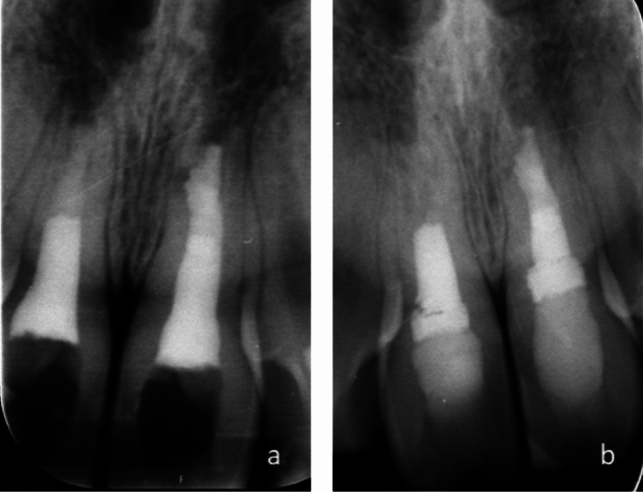
Periapical radiographs of tooth 11 and 21. **a** Tooth 11 is apexified with Biodentine™ and tooth 21 with MTA. **b** Follow-up radiograph 8 years later showing decreased radiopacity of Biodentine™ plug. Both teeth show complete healing with no periradicular radiolucency

**Fig. 2:**
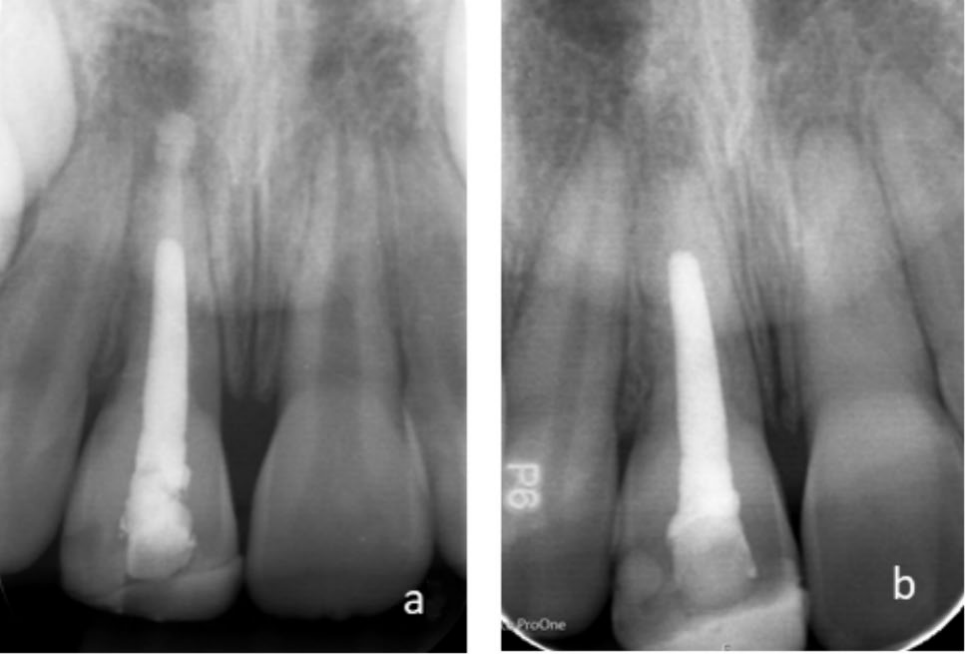
Periapical radiographs of 11. **a** Biodentine™ extruded out of apex in tooth 11. **b** The same tooth 3 years and 5 months later showing that excess Biodentine™ had resorbed, and no periradicular radiolucency

Four teeth treated with Biodentine™ (7.8%) showed reduced radiopacity of the bioceramic plug during follow-up (Fig. 1). In two teeth overfilled with Biodentine™, excess material changed radiopacity during follow-up (Fig. 2), indicating resorption of excess material.

**Fig. 3:**
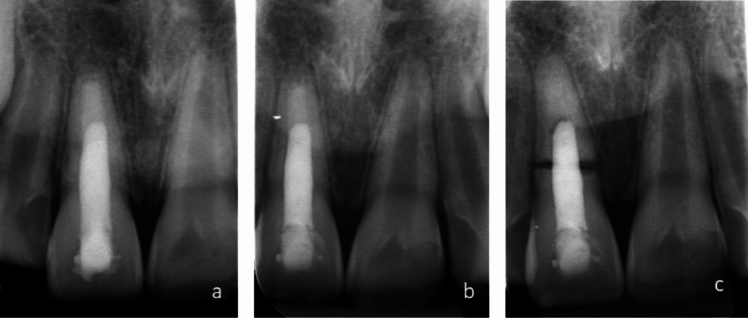
Periapical radiographs showing spontaneous cervical root fracture in a maxillary central incisor. **a** Tooth 11 at apexification with Biodentine and Gutta-percha, **b** Conditions 1-year post-treatment, **c** Spontaneous fracture discovered 19 months post-treatment

Five teeth (9.8%) with very immature root development exhibited spontaneous root fracture between 1 and 6 years after endodontic treatment (Fig. 3). Presence of root resorption at apexification or type of bioceramic were not associated with root fracture (Table 4). Teeth exhibiting spontaneous root fracture had somewhat longer treatment time with calcium hydroxide dressing than teeth without fracture, mean 14.6 (SD 11.2) vs. 7.1 (SD 7.6) month (*p* = 0.049).

**Table 4:** Root development, root resorption and type of bioceramic for all teeth and comparison of teeth with and without spontaneous root fracture (*n* = 48)

	All		Spontaneous root fracture*				
	(*n* = 51)		No (*n* = 43)		Yes (*n* = 5)		
	%	(*n*)	%	(*n*)	%	(*n*)	*P*-value
*Root development*							
Very immature	52.9	(27)	44.2	(19)	100.0	(5)	< 0.05
Immature	47.1	(24)	55.8	(24)	0	(0)	
*Root resorption*							
Present	33.3	(17)	37.2	(16)	0	(0)	ns
Absent	66.7	(34)	62.8	(27)	100.0	(5)	
*Type of bioceramic*							
Biodentine™	78.4	(49)	79.1	(34)	80.0	(4)	ns
MTA	21.6	(11)	20.9	(9)	20.0	(1)	

^*^Reduced number because of internal drop-out

## Discussion

This study aimed to evaluate outcome of immature permanent teeth after apexification with a bioceramic plug. The results showed that almost all teeth (88%) were classified as radiographically healed, which are in line with previous studies reporting success rates between 81 and 100% (Holden et al. 2008; Pace et al. 2014; Bonte et al. 2015; Ree and Schwartz 2017; Kandemir Demirci et al. 2020; Santos et al. 2022). Healing continues after placement of an apical plug, and with longer observation time increased healing outcome could be expected. One study showed increased healing from 58% at 1-year follow-up to 81% at 5 years and 94% after 10 years in teeth apexified using bioceramics (Pace et al. 2014). With longer follow-up time, we assume similar findings could be expected in the present study. In contrast, another study showed that success rate decreased from 95 to 91% after a 4-year follow-up of teeth after apexification using calcium hydroxide (Cvek 1992). Both studies state the importance of long-term follow-up.

The results showed that preoperative signs of infection influenced healing outcome. Previous studies have shown contradictory results regarding preoperative infection and healing (Mente et al. 2013; Kandemir Demirci et al. 2020). One study found that preoperative apical periodontitis and the operator’s experience were associated with outcome (Mente et al. 2013). One explanation for the non-healed teeth in the present study may be that the original infection was not eradicated, possibly due to a wide pulp space and difficulty in debridement, which in turn could lead to residues of bacteria and tissue breakdown products, preventing the healing process. The results highlight the importance of careful debridement and disinfection of immature roots before root filling.

In the present study, half of the teeth had calcium hydroxide dressing for more than 6 months and some even longer than 12 months. This is not in line with recommendations for endodontic treatment of immature teeth (Krastl et al. 2021). The reason was mainly long discussions on treatment alternatives and patient compliance. The results highlights need for information on endodontic treatment of immature teeth in the dental services.

Biodentine™ had similar success rate as MTA (85.0% vs 90.9%) in this study. However, the observed frequency of non-ideal plugs was higher in teeth treated with MTA compared to Biodentine™, which could be attributed to the poor handling characteristics and reported difficulties using MTA compared to Biodentine™ (Dawood et al. 2017).

An interesting finding was that when Biodentine™ was unintentionally extruded out of apex and into the periapical tissue, it appeared to resorb and the periradicular lesion healed (Fig. 2). Similar findings with extruded MTA are reported in other studies (Nosrat et al. 2012; Ree and Schwartz 2017). One explanation may be that extruded particles are not bound into an insoluble mass and is most likely diluted by body fluids (Ree and Schwartz 2017). Teeth with unintentionally extruded bioceramic need careful monitoring to ensure healing.

Spontaneous root fracture was observed in almost a quarter (5/24) of the very immature teeth. The results showed a lower frequency of fractures than a previous study where 40% of the immature teeth fractured after a 4-year observation period (Cvek 1992). In that study, spontaneous root fracture occurred from 77% in teeth with the most immature roots to 28% in teeth with least immature root development. Fractures were also related to cervical defects following healing from external root resorptions, which was not found in the present study. It has been speculated that calcium hydroxide reduced the hardness and modulus of elasticity of dentin which can increase risk of root fractures (Bonte et al. 2015). In the present study, four out of five teeth with spontaneous root fracture had calcium hydroxide dressing for ≥ 10 months. The extended time with calcium hydroxide may have increased the risk of fracture in addition to weak dentinal walls because of very immature root development. An in vitro study showed that there was no significant difference in fracture resistance in teeth with Biodentine™ or MTA apical plug (Yasin et al. 2021). The results support the recommendation that treatment should be completed as fast as possible to prevent risk of fracture (Krastl et al. 2021).

The results showed favourable prognosis in teeth after apexification using Biodentine™. Advantages with Biodentine™ is that apexification and root filling with GP can be performed in a single visit due to short setting time, it does not induce crown discoloration, is less expensive than MTA and have superior mechanical properties (Grech et al. 2013; Możyńska et al. 2017). However, some limitations with Biodentine™ are poor radiopacity and high washout (Grech et al. 2013). As Biodentine™ has several advantages compared to MTA, Biodentine™ can be recommended as material for apexification of non-vital immature teeth.

The strength of this study was the large number of teeth included, the study was conducted in an academic setting, adhering to a standardised protocol, and treatment supervised by experienced specialist in paediatric dentistry. Several dentists performed the treatment, which represents the clinical setting in the dental services. However, a limitation was that several postgraduate students who had limited previous experience with the apical plug technique performed treatment. This does not necessarily reflect the clinical scenario where children with non-vital immature teeth often are referred to a trained specialist in endodontics or paediatric dentistry.

PAI-score was used to evaluate periapical health and was chosen to assess outcome of apexification. It has been suggested that PAI-score is not ideal for the evaluation of immature teeth due to differences compared to mature teeth in bone reorganisation, the presence of Hertwig’s epithelial root sheet and cell differentiation seen histologically. A precise scoring of difference between 1 and 2 is, therefore, not possible in immature teeth (Simon et al. 2007). The present study used modified PAI criteria which combines score 1 and 2 and uncertainty between these scorings was avoided (Holden et al. 2008). However, since the PAI-score does not differentiate between the sizes of the lesions, periapical healing may occur without change in PAI-score. This may lead to a lower success rate, which may have influenced success rate in the present study.

## Conclusions

- Apexification of immature permanent teeth using bioceramics showed favourable prognosis
- Teeth with signs of infection before endodontic treatment need careful treatment and infection control before apexification
- Spontaneous root fracture occurred only in five teeth (< 10%) with very immature root development

## Data Availability

Data supporting this study cannot be made available due to ethical reasons.
